# Point-of-care urine Gram stain led to narrower-spectrum antimicrobial selection for febrile urinary tract infection in adolescents and adults

**DOI:** 10.1186/s12879-022-07194-9

**Published:** 2022-03-01

**Authors:** Tomohiro Taniguchi, Sanefumi Tsuha, Soichi Shiiki, Masashi Narita

**Affiliations:** 1 Division of Infectious Diseases, Department of Internal Medicine, Okinawa Chubu Hospital, 281 Miyazato, Uruma, Okinawa 904-2293 Japan; 2 Division of General Internal Medicine and Infectious Diseases, Hiroshima Prefectural Hospital, 1-5-54 Ujinakanda, Minamiku, Hiroshima 734-8530 Japan; 3 Division of General Internal Medicine and Infectious Diseases, Sakibana Hospital, 1-3-30 Nozomino, Izumi, Osaka 594-1105 Japan

**Keywords:** Point-of-care Gram stain, Urine Gram stain, Febrile urinary tract infection, Pyelonephritis, Prostatitis, Antimicrobial resistance

## Abstract

**Background:**

Febrile urinary tract infections (fUTIs), which include pyelonephritis, prostatitis, and urosepsis, are the most common cause of sepsis. However, the treatment has become more complex because of the worldwide increase in antimicrobial resistance (AMR). The objective of this study was to clarify whether point-of-care Gram stain (PCGS) of urine contributed to fUTI diagnosis and treatment in adults.

**Methods:**

This hospital-based observational study was undertaken between January 2013 and March 2015 in Okinawa, Japan. All enrolled patients were adults who had been admitted to the Division of Infectious Diseases with suspected fUTI. The usefulness of PCGS results were compared for urinalysis (U/A) and urine cultures (U/Cs). The targeted therapy type and its susceptibility based on PCGS were analyzed, and each was investigated in two groups: the uncomplicated pyelonephritis group and the complicated pyelonephritis/prostatitis group.

**Results:**

Two hundred and sixty-six patients were enrolled. The results of PCGS were closely correlated with those of U/A for pyuria and bacteriuria, and moderately correlated with the results of U/C for bacterial types. In the uncomplicated group, narrow-spectrum antimicrobials such as cefotiam were initially selected in 97.9% (47/48) of patients, and their susceptibility was 97.9% (47/48). In the complicated group, the susceptibility was 84.2% (186/221) (*p* = 0.009) despite frequent AMRs (14.7%; 32/218) and low use of broad-spectrum antimicrobials such as carbapenems (7.7%; 17/221).

**Conclusion:**

Urine PCGS led to a more precise fUTI diagnosis and prompted clinicians to select narrower-spectrum antibiotics with high susceptibility.

**Supplementary Information:**

The online version contains supplementary material available at 10.1186/s12879-022-07194-9.

## Background

Febrile urinary tract infections (fUTIs), which include acute pyelonephritis, prostatitis, and urosepsis, can cause sepsis, septic shock, and death [1, 2]. Febrile UTI diagnosis is not straightforward [3], and the treatment has become more complicated due to the increasing antimicrobial resistance (AMR) [2, 4]. Ceftriaxone, a third-generation cephalosporin, is commonly recommended as an empirical treatment for uncomplicated pyelonephritis, while carbapenems are recommended for complicated pyelonephritis because of the concern about an increase in extended-spectrum beta-lactamases (ESBLs) [5].

Gram staining in clinical microbiology assists in diagnosing fUTI, while evidence for the fUTI treatment strategy is limited [3, 6]. Point-of-care Gram stain (PCGS) performed by clinicians has reduced broad-spectrum antimicrobial overuse at Okinawa Chubu Hospital (OCH) in Japan [7]. Urine PCGS has been used here in children [8]. However, the usefulness of PCGS in diagnosing and treating fUTI in adults has never been investigated, and its clinical impact remains unclear.

The objective of this study was to clarify whether urine PCGS contributed to etiologic agent estimation and targeted treatment of fUTI in adults. In addition, fUTI patients were divided into uncomplicated and complicated groups because patients with complicated pyelonephritis are more likely to have AMR [9]. For each group, we investigated whether PCGS detected pyuria and bacteriuria, estimated bacterial types, and promoted narrower-spectrum antimicrobial use and effective treatment. On the basis of our findings, we recommend using urine PCGS to ensure a more precise fUTI diagnosis and to select narrower-spectrum antimicrobial agents.

## Methods

### Study design and setting

This was a single hospital-based, retrospective observational study. The study setting was OCH, Japan. Approximately 39,000 patients visit the emergency room (ER) annually and nearly 7,000 patients are hospitalized after attending the ER each year [10]. Most patients with a suspected fUTI are initially examined in the ER. Adolescent and adult patients who are diagnosed with fUTI and who need to be hospitalized are admitted to the Division of Infectious Diseases. Patients with complicated pyelonephritis requiring surgical interventions such as double-J stent insertion, suprapubic cystostomy, or nephrostomy are admitted to the Department of Urology.

### Case definitions and data collection

Febrile UTI was considered if a patient had symptoms of systemic inflammation (fever, chills, and malaise) and bladder symptoms (urinary frequency, urgency, and dysuria), which was supported by urinalysis (U/A) results that showed pyuria or bacteriuria (or both) and urine culture (U/C) results that showed substantial concentrations of a uropathogen [1]. Febrile UTI patients were divided into the following two groups: the uncomplicated group, which included patients with uncomplicated pyelonephritis; and the complicated group, which included patients with complicated pyelonephritis and prostatitis. Uncomplicated pyelonephritis was defined as acute pyelonephritis in women who did not have any underlying diseases and were not pregnant [11]. Complicated pyelonephritis was defined as acute pyelonephritis in patients who had underlying diseases such as neurogenic bladder, indwelling bladder catheter, diabetes mellitus, or prostate hypertrophy, or patients whose kidney had urolithiasis, abscess, or emphysematous pyelonephritis/cystitis. Undefined cases were categorized into the complicated group [11]. Prostatitis was defined as fUTI without flank pain, with prostate tenderness, or a significant increase in prostate-specific antigen (PSA) or an imaging diagnosis [9, 12].

In this study, all patient information was collected from medical charts between January 2013 and March 2015. The inclusion criteria were all patients aged 15 years or older who were admitted to the Division of Infectious Diseases and finally diagnosed with UTI. The exclusion criteria were as follows: (1) uncomplicated cystitis; (2) previous antibiotics exposure within 48 h; (3) PCGS not examined or U/C not submitted; (4) not diagnosed with a fUTI after admission; (5) patients with co-infection other than fUTI; (6) transferred to another hospital from the ER; and (7) data insufficient.

### Urinalysis and urine culture

Clean-voided midstream urine or urine collected with a disposable catheter was used for PCGS in the ER. For patients with indwelling urinary catheters, urine was collected directly from the catheter rather than from the urine bag. The residue specimen was then delivered to the laboratory for U/A and U/C. If catheter-associated urinary tract infection was suspected, the catheter was replaced. U/A was performed after centrifugation without staining by a technician and using an automated urine analyzer (Aution Hybrid, Arkray, Kyoto, Japan). The pyuria result was considered to be positive when five or more leukocytes per 400 × magnification field (high power field: HPF) were observed, and the number of leukocytes was quantified. The bacteriuria result was considered to be positive if an average of one or more bacteria was identified in a HPF of view, and the number of bacteria was semi-quantified as follows: 1 + , an average of one or more in one visual field; 2 + , many in one visual field; and 3 + , a large number in one visual field.

U/C was performed by a technician using uncentrifuged urine. The number of bacteria was quantified using quantitative medium (Caldip Plus, Merck, Darmstadt, Germany). If the urine sample amount was insufficient, this quantification was omitted, but semi-quantification was confirmed using culture plates. If Gram-positive rods such as *Bacillus* or *Corynebacterium*, coagulase-negative staphylococci other than *Staphylococcus lugdunensis* or *Staphylococcus saprophyticus*, or other skin or vaginal normal flora grew in the culture, it was considered to be contaminated and non-pathogenic.

Enterobacteriaceae with ESBL-producing or AmpC-positive or carbapenem-resistant, carbapenem-resistant *Pseudomonas aeruginosa*, methicillin-resistant *Staphylococcus aureus*, and vancomycin-resistant *Enterococcus* were considered to indicate AMR.

### Blood culture

At least two sets of blood cultures, which included aerobic and anaerobic bottles with Bactec Plus resin medium (Becton, Dickinson and Company, Franklin Lakes, NJ, USA), were taken from the upper or lower limbs but not from the femoral vessels. All bottles were incubated for at least 5 days using the Bactec 9240 system, which is an automated blood culture system [13]. Coagulase-negative staphylococci, *Bacillus*, *Propionibacterium*, *Micrococcus*, *Clostridium*, and α-streptococci were considered to be potential skin contaminants [13]. With the exception of α-streptococci, if any of these grew in only one set of blood cultures, it was considered to be a contaminant.

### PCGS and antimicrobial selection

PCGS was performed and interpreted by physicians in the ER soon after urine samples were obtained. Uncentrifuged urine specimens were placed on glass slides, fixed with a flame or hot air blower, and Gram-stained using Barmii M (Muto Pure Chemicals, Tokyo, Japan). We used a four-step dyeing procedure including crystal violet, 2% iodine sodium hydroxide, acetone ethyl alcohol, and 0.1% fuchsine. The slides were examined in the ER by in-house staff members including trained resident physicians (postgraduate years 1 and 2, called junior residents) to identify each etiologic agent and select an appropriate targeted antimicrobial therapy [7, 14]. Physicians examined the slides using conventional light microscopy at 100 × magnification and then at 1000 × magnification under immersion oil. If leukocytes were visible, the patient was considered to have pyuria, and the number of leukocytes was semi-quantified as follows: an average of < 1 in one per visual field (1000 ×) was ± ; an average of 1 to 9 was 1 + ; 10 to 99 was 2 + ; and > 99 was 3 + . Similarly, if bacteria were present, the patient was considered to have bacteriuria, and the number of bacteria was semi-quantified as follows: an average of < 1 per visual field (1000 ×) was ± ; an average of 1 to 9 was 1 + ; 10 to 99 was 2 + ; and > 99 was 3 + . All findings were confirmed by senior residents (postgraduate years 3 to 5) and attending physicians. If the junior residents’ interpretation differed from that of senior residents or attending physicians, direct feedback to the junior residents was given from an educational viewpoint.

PCGS has been used for a practical and useful diagnostic tool since 1976 at OCH [7]. All junior residents in the first year rotate to the division of infectious diseases for 2 weeks and receive basic education on infectious diseases. In addition, junior residents of internal medicine or primary care in the second year rotate to the division for over a month, as do senior residents in their elective months. All attending physicians in the division also received education on infectious diseases as alumni of the internal medicine residency program of OCH.

Annual cumulative antimicrobial susceptibility test data were reported and distributed to medical staff by the bacteriology laboratory. The organisms that were estimated using PCGS led physicians to select antibiotics along with the cumulative antibiogram. For example, 1415 cultures were positive for *Escherichia coli* in 2015, the sensitivity of which to ampicillin was 62%, ampicillin/sulbactam 69%, cefotiam (a second-generation cephalosporin, alternative to cefuroxime in Japan) 82%, cefmetazole (a cephamycin, alternative to cefoxitin in Japan) 99%, cefotaxime 84%, tobramycin 94%, and ciprofloxacin 79%. ESBL strains accounted for 16% of the bacteria cultured. In another example, 404 cultures were positive for *P. aeruginosa*, and the sensitivity to piperacillin was 95%, while that of ceftazidime was 96%, aztreonam 91%, imipenem/cilastatin 95%, tobramycin 99%, and ciprofloxacin 93%. During this study, the cumulative antibiogram was updated yearly from 2013 to 2015.

In urine PCGS, medium- or large-sized Gram-negative rods suggested *E. coli*, *Proteus mirabilis*, or *Klebsiella pneumoniae*. In these cases, cefotiam 1 g every 6 to 8 h (q6–8 h) intravenously (iv) was mostly administered. A third-generation cephalosporin (cefotaxime 1 g q6–8 h iv or ceftriaxone 1–2 g q12–24 h iv) was selected when *Citrobacter*, *Enterobacter*, or other Enterobacteriaceae was suspected based on previous culture findings. If an ESBL-producing bacterial strain was suspected based on a living place or previous culture results, cefmetazole 1 g q6–8 h iv was selected. If a patient’s condition was unstable due to septic shock, a carbapenem (meropenem 1 g q8h iv or imipenem/cilastatin 0.5 g q6h iv) was used. Small-sized Gram-negative rods suggested *P. aeruginosa*, ceftazidime 1 g q6–8 h iv, aztreonam 1 g q6–8 h iv, tobramycin 120–240 mg q24h iv, or a carbapenem (meropenem or imipenem/cilastatin) was chosen. Gram-positive cocci in chains suggested *Enterococcus* or *Streptococcus*, and mainly ampicillin 1 g q6–8 h iv or rarely vancomycin 1 g q12h was selected. Gram-positive cocci in clusters suggested *Staphylococcus* or *Aerococcus*, and cefazolin 1 g q6–8 h iv or rarely vancomycin was chosen. If Gram-negative rods and Gram-positive cocci in chains were observed, ampicillin/sulbactam 1.5 g q6–8 h iv was chosen to cover both Enterobacteriaceae and *Enterococcus*. When more than two types of bacteria were confirmed, a polymicrobial infection including AMR was suspected, and a cephalosporin, a cephamycin, or a carbapenem was selected. These polymicrobial infection cases were excluded from the analysis in Table 3, because they were unevaluable on PCGS. If Gram-positive rods, small amounts of Gram-positive cocci or mixed organisms were observed, they were considered to be non-pathogenic.

Penicillins and first- or second-generation cephalosporins were defined as narrow-spectrum antibiotics; fourth-generation cephalosporin, carbapenems, and vancomycin as broad-spectrum antibiotics; and all other antibiotics as intermediate-spectrum antibiotics [7].

### Outcome measures

The primary outcome was the susceptibility of targeted therapies such as initial antimicrobial selection based on PCGS according to urine and/or blood culture susceptibility test results in the uncomplicated and complicated groups. Secondary outcomes were pyuria and bacteriuria detection by PCGS compared to U/A, and bacterial type estimation by PCGS compared to U/C.

### Statistical analysis

To ensure an adequate sample size to calculate the susceptibility of the initial antimicrobial choice, we hypothesized that there would be a difference between the uncomplicated and complicated groups. We assumed that the rate of susceptibility in the uncomplicated group would be more than 0.9 because the patients were young and the rate of AMR was still low. In the complicated group, however, we expected that the susceptibility could be lower than 0.9 because they were older, and repeated antimicrobial exposure increases AMR colonization. Therefore, assuming that the rate of susceptibility would be 0.95 in the uncomplicated group and 0.8 in the complicated group, and assuming that the uncomplicated group-to-complicated group ratio was 1:4, 80% power, and a two-sided alpha level of 0.05, we calculated that 260 patients would be required.

The Chi-squared or Fisher’s exact test was used for categorical variables, and the Student’s *t*-test was used for numerical variables. Cuzick’s test for trend was used to compare PCGS and U/A results. Kappa statistics were used to evaluate the agreement between PCGS and U/C results. Additionally, *p* < 0.05 was considered to be significant. The statistical analysis was performed using Stata software (version 16.1; StataCorp, College Station, TX, USA).

## Results

During the study period, 502 patients were enrolled, and 236 patients were excluded (138 other co-infection, 69 previous antimicrobial exposure, 9 not fUTI, 8 data insufficient, 6 transferred, 2 PCGS not examined, 2 U/C not submitted, and 2 uncomplicated cystitis).

There were 48 and 218 patients enrolled in the uncomplicated and complicated groups, respectively. Table 1 shows the comparison between the two groups. Patient age ranged from 16 to 92 years in the uncomplicated group and from 19 to 105 years in the complicated group. The complicated group included 34 patients with prostatitis, ten with stone pyelonephritis, three with uterine prolapse, two with ileal conduit, two with renal abscess, one with emphysematous cystitis, and one with vesicoureteral reflux. The performance status was classified in accordance with the Eastern Cooperative Oncology Group scale [15].

**Table 1:** Comparison between uncomplicated and complicated groups

	Uncomplicated group (N = 48)	Complicated group (N = 218)	*p* value
Median age (IQR)	41 (31–60)	80 (69–88)	< 0.0001*
Female sex	48 (100%)	139 (63.8%)	< 0.001*
Performance status			
0–2	48 (100%)	144 (66.0%)	< 0.001*
3–4 (in bed more than 50%)	0	74 (33.9%)	< 0.001*
Living place before admission			
Home	48 (100%)	156 (71.6%)	< 0.001*
Nursing facility or hospital	0	62 (28.4%)	< 0.001*
Symptom duration days (IQR)	2 (1–3)	1 (0–2)	0.0065*
Bladder inflammatory symptoms	36 (75.0%)	73 (33.5%)	< 0.001*
Shaking chills	35 (72.9%)	85 (39.0%)	< 0.001*
Physical examinations			
CVA tenderness	38 (79.2%)	46 (21.1%)	< 0.001*
Bimanual palpation	38 (79.2%)	48 (22.0%)	< 0.001*
Prostate tenderness	0	20 (27.8%)	0.029*
Catecholamine support needed	1 (2.1%)	7 (3.2%)	1.000
Urinalysis			
Pyuria	46 (95.8%)	215 (99.1%)	0.15
Bacteriuria	47 (97.9%)	212 (97.7%)	0.70
Urine PCGS			
Pyuria	48 (100%)	215 (98.6%)	0.55
Bacteriuria	45 (93.8%)	213 (97.7%)	0.16
Urine culture results	*Escherichia coli* 44	*Escherichia coli* 157	
	*Enterobacter aerogenes* 3	*Klebsiella* spp. 24	
	*Streptococcus* spp. 2	*Pseudomonas aeruginosa* 12	
	Nonpathogenic 1	*Citrobacter* spp. 11	
		*Streptococcus* spp. 11	
		*Proteus mirabilis* 9	
		*Enterococcus faecalis* 6	
		*Morganella morganeii* 6	
		*Serratia marcescens* 4	
		Others 10	
		Nonpathogenic 11	
		Negative 1	
Blood culture positivity	8 (16.7%)	67 (30.7%)	0.053
Blood culture results	*Escherichia coli* 8	*Escherichia coli* 57	
		*Klebsiella* spp. 5	
		*Enterococcus* spp. 2	
		Others 6	
Antimicrobial resistance	1 (2.1%)	32 (14.7%)	0.014*
Initial antibiotics			
Based on PCGS	45 (93.8%)	184 (84.4%)	0.064
Based on previous cultures	1 (2.1%)	31 (14.2%)	0.010*
Empirical treatment	2 (4.1%)	3 (1.4%)	0.22
Death	0	2 (0.9%)	0.51

*CVA* costovertebral angle

*IQR* interquartile range

*PCGS* point-of-care Gram stain

*p < 0.05

Nearly two-thirds of the patients (36/48) in the uncomplicated group and only one-third (73/218) of patients in the complicated group had at least one bladder symptom, such as dysuria, urinary frequency, and urinary urgency.

The physical examination results showed that the frequency of costovertebral angle (CVA) tenderness and bimanual palpation of the upper abdomen was nearly equal in both groups. The location of CVA tenderness was 63.2% (24/38) on the right side, 31.6% (12/38) on the left side, and 5.3% (2/38) on an unknown side in the uncomplicated group. In the complicated group, CVA tenderness was located on the right side in 37.0% (17/46) of patients, left side in 28.3% (13/46), both sides in 13.0% (6/46), and unknown side in 21.7% (10/46). Prostate tenderness was positive in 58.9% (20/34) of patients with prostatitis.

U/C results in the uncomplicated group for 44 *E. coli* included 37 ampicillin-sensitive (84.1%) and one ESBL-positive (2.3%) results. Two *Streptococcus* spp. included *S. agalactiae* and group F *Streptococcus*.

U/C results in the complicated group for 157 *E. coli* included 87 ampicillin-sensitive (55.4%) and 26 ESBL-positive (16.6%) results. Twenty-four *Klebsiella* results included 22 K*. pneumoniae* and two *K. oxytoca* results, but no ESBL. Twelve *Pseudomonas* spp. included one carbapenem-resistant *P. aeruginosa* and one *P. alcaligenes*. Eleven *Streptococcus* spp. included four *S. agalactiae*, three *S. dysgalactiae*, one *S. oralis*, one *S. alactolyticus*, and two other α-streptococcus. Eleven *Citrobacter* spp. included two *C. koseri* AmpC positives. Nine *P. mirabilis* included one ESBL positive. Ten others included three *Providencia stuartii*, two *Myroides* spp., two *S. aureus*, and one each of *Aerococcus urinae*, *Enterobacter cloacae*, and *Sphingomonas paucimobilis*. The two *S. aureus* cultures included one methicillin-sensitive and one methicillin-resistant *S. aureus* result.

Among the blood cultures, eight *E. coli* included seven ampicillin-susceptible (87.5%) results, but there were no ESBLs in the uncomplicated group. In the complicated group, 57 *E. coli* included 34 ampicillin-sensitive (59.6%) and seven ESBL-positive (12.3%) results. Five *Klebsiella* included one AmpC-positive result. Two *Enterococcus* results were *E. faecalis* and *E. faecium*. Six other results were *Anaerococcus prevotii*, *C. koseri*, ESBL-positive *P. mirabilis*, *P. stuartii*, carbapenem-resistant *P. aeruginosa*, and *S. dysgatactiae*.

Two patients died. One had been admitted to another hospital and treated with multiple antibiotics. Soon after discharge, he developed fever with shock, and was transferred to our hospital. His Foley catheter was obstructed with debris, and he was diagnosed as having obstructive pyelonephritis with hydronephrosis. Ceftriaxone, meropenem, and vancomycin with catecholamine were administered, but he died 2 days later. Both carbapenem-resistant *P. aeruginosa* and *S. dysgalactiae* subsp. *dysgalactiae* were cultured from the patient’s blood and urine. The other patient who died was a steroid user with *E. coli* urosepsis. Her fUTI had resolved, but she developed cytomegalovirus gastritis and it eventually caused her death after 3 weeks.

Table 2 compares pyuria and bacteriuria detection by PCGS with U/A. In the complicated group, U/A was not submitted for one patient who was not included in the analysis (N = 217). As the number of white blood cells in the U/A results increased, the number of white blood cells in PCGS tended to increase in both the uncomplicated and complicated groups (each *p* < 0.001). Similarly, as the number of bacteria in the U/A results increased, the number of bacteria in PCGS tended to increase in both groups (each *p* < 0.001).

**Table 2:** Pyuria and bacteriuria detection by PCGS and U/A

Uncomplicated group (N = 48)						
	U/A WBC 1–4	5–9	10–29	30–49	50–100/HPF	Total
PCGS WBC						
0	0	0	0	0	0	0
±	0	0	0	0	0	0
1 +	2	2	8	0	3	15
2 +	0	0	4	6	13	23
3 +	0	0	0	2	8	10
Total	2	2	12	8	24	

	U/A bacteria 0	±	1 +	2 +	3 +	Total
PCGS bacteria						
0	0	0	2	1	0	3
±	1	0	4	3	0	8
1 +	0	0	2	3	4	9
2 +	0	0	2	7	13	22
3 +	0	0	0	3	3	6
Total	1	0	10	17	20	

Complicated group (N = 217)						
	U/A WBC 1–4	5–9	10–29	30–49	50–100/HPF	Total
PCGS WBC						
0	1	1	1	0	0	3
±	1	4	12	7	3	27
1 +	0	3	17	21	42	83
2 +	0	0	4	7	88	99
3 +	0	0	0	0	5	5
Total	2	8	34	35	138	

	U/A bacteria 0	±	1 +	2 +	3 +	Total
PCGS bacteria						
0	2	1	1	1	0	5
±	0	1	15	6	1	23
1 +	3	0	10	10	5	28
2 +	0	1	16	32	63	112
3 +	0	0	2	10	37	49
Total	5	3	44	59	106	

*p* < 0.001

*HPF* high power field

*PCGS* point-of-care Gram stain

*U/A* urinalysis

*WBC* white blood cell

In Table 2, pyuria detection by U/A was 95.8% (46/48), while that by PCGS was 100% (48/48) in the uncomplicated group (*p* = 0.495). In the complicated group, pyuria detection by U/A was 99.1% (215/217), while that by PCGS was 98.6% (214/217) (*p* = 1.000). The only patient whose pyuria was negative by both U/A and PCGS had the complication of water intoxication. The day after water restriction was introduced, over 100/HPF leukocytes were detected using U/A, and 10^4^ colony-forming units/mL (CFU/mL) of *E. coli* was cultured in the urine.

Bacteriuria detection by U/A was 97.9% (47/48), while that by PCGS was 93.8% (45/48) in the uncomplicated group (*p* = 0.617). In the complicated group, bacteriuria detection by U/A was 97.7% (212/217), while that by PCGS was 97.7% (212/217; *p* = 1.000) (Table 2). The two patients who had negative bacteria results for both U/A and PCGS were the above-mentioned patient with water intoxication and another who had taken sulfamethoxazole/trimethoprim 3 weeks before because of cystitis, and 10^3^ CFU/mL of *E. coli* was cultured from this patient’s urine.

Table 3 shows the agreement between bacterial estimation using PCGS and U/C. In the uncomplicated group, the U/C results for one patient were considered to be a skin or vaginal contaminant, and this patient was excluded from the analysis of Tables 3 and 4. The agreement was 90.2%, and the kappa coefficient was 0.518 (95% confidence interval [CI] 0.318–0.547). The sensitivity to detect Enterobacteriaceae was 93.6%, and the specificity was 100% using PCGS.

**Table 3:** Agreement between bacteria estimation using urine PCGS and urine culture results

Uncomplicated group (N = 47)			
	Urine culture		
	Enterobacteriaceae	*Streptococcus*	Nonpathogenic
Urine PCGS			
GNR middle or large size	44	0	0
GPC in chains	0	0	0
Nonpathogenic	0	0	2
Negative	3	2	0
Kappa (95% CI) = 0.518 (0.318–0.547)			

Complicated group (N = 194)						
	Urine culture					
	Enterobacteriaceae	*Pseudomonas*	*Streptococcus* or *Enterococcus*	*Staphylococcus* or *Aerococcus*	Nonpathogenic	Negative
Urine PCGS						
GNR middle or large size	167	5	0	0	1	0
GNR small size	4	5	0	0	1	2
GPC in chains	0	0	7	0	2	10
GPC in clusters	0	0	0	2	1	2
Nonpathogenic	0	0	1	0	6	0
Negative	4	0	0	1	0	1
Kappa (95% CI) = 0.608 (0.571–0.653)						

*CI* confidence interval

*GNR* Gram-negative rods

*GPC* Gram-positive cocci

*PCGS* point-of-care Gram stain

In the complicated group, the U/C results of five patients were considered to be contaminated, and these results were excluded from Tables 3 and 4. The agreement was 85.8%, and the kappa coefficient was 0.608 (95% CI 0.571–0.653). The sensitivity of PCGS to detect Enterobacteriaceae was 95.4% and the specificity was 87.2%.

**Table 4:** Comparison between bacterial PCGS semi-quantification and urine culture quantification

Uncomplicated group (N = 47)					
	Urine culture				
	10^4^	10^5^	10^6^	10^7^ CFU/mL	Total
PCGS bacteria					
0	1	1	1	0	3
±	2	5	0	1	8
1 +	1	0	1	6	8
2 +	0	1	4	17	22
3 +	0	0	0	6	6
Total	4	7	6	30	

Complicated group (N = 213)							
	Urine culture						
	Negative	10^3^	10^4^	10^5^	10^6^	10^7^ CFU/mL	Total
PCGS bacteria							
0	1	1	1	0	0	2	5
±	0	4	4	3	3	7	21
1 +	0	0	4	8	4	11	27
2 +	0	1	1	5	8	96	111
3 +	0	0	1	2	2	44	49
Total	1	6	11	18	17	160	

*p*<0.001

CFU, colony-forming units

PCGS, point-of-care Gram stain

As mentioned above, 19 patients with PCGS results that showed more than two types of bacteria were excluded from the analysis in Table 3. Gram-negative rods such as Enterobacteriaceae, *Pseudomonas*, and *Myroides*, Gram-positive cocci such as *Enterococcus* and *Streptococcus*, and Gram-positive rods such as *Corynebacterium* or others were cultured from these patients.

There were seven discrepancies in PCGS interpretation between trained young resident physicians and attending physicians. Correct interpretations were found in one of the residents and four of the attending physicians. Both made misinterpretations in one case, and one case was deemed unevaluable.

Table 4 shows the comparison between PCGS bacterial semi-quantification and U/C bacterial quantification. Contamination was suspected in one patient in the uncomplicated group and five patients in the complicated group, and they were excluded from this table. As the number of cultured bacteria increased, the PCGS semi-quantified bacteria increased in both groups (each *p* < 0.001). There was one patient with a negative urine culture result in the complicated group. This patient was diagnosed with acute prostatitis, and *K. pneumoniae* was identified in the blood culture. After treatment, his PSA level decreased from 33 to 7 ng/mL.

Table 5 shows the susceptibility of initially chosen antibiotics based on the U/C results. Three patients received combination therapy in the complicated group. Narrow-spectrum antimicrobials were initially chosen in 97.9% (47/48) in the uncomplicated group and 76.9% (170/221) in the complicated group (*p* < 0.001). Broad-spectrum antibiotics were selected in 7.7% (17/221) of patients in the complicated group only. The overall susceptibility was 97.9% (47/48) in the uncomplicated group and 84.2% (186/221) in the complicated group (*p* = 0.009). Of the 15 patients who used empiric carbapenems, 5 had stone pyelonephritis, 5 had a history of ESBL detection, 4 had septic shock, and 1 had a recent hospitalization.

**Table 5:** Initial antibiotic susceptibility

	Uncomplicated group (N = 48)	Complicated group (N = 218)	*p* value
Narrow spectrum	46/47 (97.9%)	142/170 (83.5%)	< 0.001*
Ampicillin		5/5	
Ampicillin/sulbactam		1/3	
Cefazolin		2/2	
Cefotiam	46/47	134/160	
Intermediate spectrum	1/1 (100%)	29/34 (85.3%)	1.000
Cefmetazole		10/12	
Cefotaxime or ceftriaxone	1/1	11/13	
Ceftazidime		5/5	
Aztreonam		2/3	
Tobramycin		1/1	
Broad spectrum	0	16/17 (94.1%)	
Imipenem/cilastatin		8/8	
Meropenem		6/7	
Vancomycin		2/2	
Total	47/48 (97.9%)	186/221 (84.2%)	0.009*

*p < 0.05

Table 6 shows the finally chosen iv antibiotics after the U/C results were reported. Combination antimicrobials were selected for three patients. Narrow-spectrum antibiotics were chosen in 91.7% (44/48) of patients in the uncomplicated group and 67.8% (150/221) in the complicated group (*p* = 0.001). Ampicillin, the narrowest-spectrum antimicrobial in this study, was finally selected in 42.4% (114/269). Broad-spectrum antibiotics were finally used in 7.8% (17/218) of patients in the complicated group only. Among 18 cefmetazole cases, ESBL-producing *E. coli* were cultured from 17 patients. One patient also had positive blood culture results.

**Table 6:** Final antibiotic administered intravenously after urine culture results

	Uncomplicated group (N = 48)	Complicated group (N = 218)
Narrow spectrum	44 (91.7%)	150 (67.8%)
Ampicillin	34	80
Ampicillin/sulbactam		1
Cefazolin		4
Cefotiam	10	65
Intermediate spectrum	4 (8.3%)	54 (24.4%)
Cefmetazole	1	17
Cefotaxime or ceftriaxone	2	10
Ceftazidime		4
Aztreonam	1	5
Tobramycin		15
Ciprofloxacin		3
Broad spectrum	0	17 (7.7%)
Cefepime		3
Imipenem/cilastatin		7
Meropenem		4
Vancomycin		3

## Discussion

Unlike previous studies on urine Gram staining in a laboratory [3, 6, 16–19], this observational study investigated the utility of urine PCGS in estimating etiologic agents and choosing narrower-spectrum antimicrobials to treat fUTI in adults. Our results yielded three main findings. First, PCGS and U/A were good indicators of both pyuria and bacteriuria. Second, estimated bacterial types using PCGS were moderately correlated with U/C results. Third, initial antibiotic susceptibility was quite high in the uncomplicated group although they were almost all narrow-spectrum antimicrobials, and the susceptibility was also high in the complicated group although there was a high level of AMR and low usage of broad-spectrum antimicrobials.

Pyuria and bacteriuria detection by PCGS and U/A showed similar results in both groups. U/A and PCGS both showed no false-negative results for pyuria and bacteriuria in the uncomplicated group. In the complicated group, there was only one patient with water intoxication, and she did not have pyuria or bacteriuria (0.46%). U/A samples were centrifuged before analysis, and thus, the sensitivity for bacterial detection may be higher than using PCGS. However, U/A was unable to distinguish between Gram-positive and -negative bacteria, and it was impossible to distinguish between pathogenic Enterobacteriaceae and non-pathogenic normal flora. PCGS does not require centrifugation, and it easily identified Gram-positive and -negative bacteria, with a small risk of technical error in the process of a four-step dyeing procedure. Additionally, especially for fUTI in the complicated group, a clinical diagnosis is often not straightforward because the patients’ symptoms and physical examination are not reliable [3]. Therefore, using both U/A and PCGS together to complement each other is strongly recommended for a precise fUTI diagnosis.

Estimating the bacterial types using PCGS was moderately correlated with U/C results in the uncomplicated group. Most Enterobacteriaceae were detected with PCGS except for three false negative results. The bacteria quantity in these false negative samples were relatively low: 10^4^, 10^5^, and 10^6^ CFU/mL. However, all Enterobacteriaceae levels that were greater than 10^6^ CFU/mL were detected by PCGS. PCGS did not detect the two *Streptococcus* results, but both of the patients grew Enterobacteriaceae together with *Streptococci*, and we considered the latter to be contaminants. Additionally, PCGS precisely identified non-pathogenic bacterial infections. Therefore, PCGS detected most pathogens unless the bacterial quantity was too low, and this led to selecting narrow-spectrum antibiotics.

In the complicated group, various types of bacteria were detected with PCGS, and identified with U/C. AMRs were more frequently found, and so it was not easy to choose the initial antimicrobial treatment. PCGS did not identify four cases of Enterobacteriaceae that grew in the cultures. The quantity of these bacteria was 10^3^, 10^4^, and two results of 10^7^ CFU/mL; the latter two culture results suggested that technical errors had occurred during the staining process. In these two cases, PCGSs were repeated using the centrifuged residues, and leukocytes and Gram-negative rods were found in both cases. The sensitivity of estimating *Pseudomonas* based on PCGS was only 50% (5/10), which would be unreliable for differentiating *Pseudomonas* from Enterobacteriaceae. However, the sensitivity for detecting Gram-negative rods including Enterobacteriaceae and *Pseudomonas* was 97.8% (181/185). When only Gram-negative rods were confirmed using PCGS, an antibiotic that excluded coverage of Gram-positive bacteria was chosen. For *Streptococcus* and *Staphylococcus*, the sensitivity was 87.5% (7/8) and 66.7% (2/3), respectively. Non-pathogenic bacteria were moderately detected using PCGS (54.5%; 6/11). Compared to other reports about Gram-positive cocci in clusters [20, 21], *S. saprophyticus* was not detected, but instead, *S. aureus* and *A. urinae* were cultured in this study.

Our study suggested that at least 10^4^ CFU/mL or more was required to make a diagnosis in the uncomplicated group, and 10^3^ CFU/mL was required for the complicated group. These results were similar to the recommendations of the Infectious Diseases Society of America (IDSA) guidelines in 1992, which recommended that 10^4^ CFU/mL or more is required to meet the diagnostic criteria of uncomplicated pyelonephritis, and 10^3^ or more is required for a complicated UTI in men [11]. In 2018, another report recommended that 10^4^ or more CFU/mL of a uropathogen is an acceptable diagnostic criterion for pyelonephritis in adults [1]. However, as mentioned above, we had one case of acute prostatitis that had a negative U/C result. His initial U/A detected only five to nine leukocytes per HPF, and the day after starting treatment, the leukocytes increased to 30 to 49 per HPF. Therefore, the initial U/C result may not be sufficient because of a small amount of pathogen even for a rare case of fUTI in men.

Initial targeted therapy susceptibility based on PCGS was very high (97.9%) in the uncomplicated group. In the IDSA guidelines, intravenous fluoroquinolone is recommended for therapy in patients with pyelonephritis who require hospitalization [22]. However, if the prevalence of fluoroquinolone resistance exceeds 10%, intermediate-spectrum antimicrobials such as ceftriaxone or aminoglycoside are recommended for initial treatment [22]. In Japan, unfortunately, the prevalence of fluoroquinolone-resistant *E. coli* was already 38.0% in 2015 [23]. Therefore, second-generation cephalosporins such as cefotiam, not fluoroquinolone, were mostly selected at OCH, and they were sufficient to treat patients in the uncomplicated group. Ceftriaxone, a third-generation cephalosporin, is more convenient than cefotiam because it is usually administered once daily. However, “it is better to use narrower-spectrum antibiotics” is one of our principles for antimicrobial selection that prefers cefotiam to ceftriaxone. The only pathogen for which cefotiam was not effective was ESBL-producing *E. coli*, and even ceftriaxone did not cover it. If the number of ESBL cases continues to increase in the future, cefmetazole [23] or cefoxitin, which are potentially effective against ESBL, will be required even for uncomplicated pyelonephritis.

In the complicated group, the initial antibiotic susceptibility was lower than in the uncomplicated group because of the greater prevalence of AMR. Narrow-spectrum antimicrobials were selected for 76.9% of patients, and its susceptibility was 83.5%, which was mainly influenced due to ESBLs. PCGS reduced broad-spectrum antimicrobial use to 7.7%, but one patient died due to *P. aeruginosa* that was resistant to ceftazidime, aztreonam, imipenem/cilastatin, tobramycin, and ciprofloxacin. In Japan, national surveillance reported that the prevalence of carbapenem-resistant Enterobacteriaceae was only 0.1%–0.2%, but that of carbapenem-resistant *P. aeruginosa* was 13.1%–18.8% in 2015 [24]. If the prevalence of AMR does not decrease, empirical combination therapy that covers various resistant patterns will be required more often in accordance with the local AMR prevalence. PCGS can potentially identify the bacterial type even in the complicated group, and it can reduce broad-spectrum antibiotic overuse, which will prevent an increase in AMR.

Ampicillin was the most frequently used final antibiotic in both groups. The most common fUTI pathogen was still ampicillin-sensitive *E. coli*. The overall proportion of AMR was 12.4% (33/266) in this study, which maintained low use of empirical broad-spectrum antibiotics (6.4%; 17/266).

This research has some limitations. First, this was a retrospective chart review study, and some information was missing. Second, our strategy is not directly applicable to other hospitals where clinicians are not familiar with Gram staining. PCGS is not a special technique, but it still requires a certain measure of training and experience. Third, our targeted therapies based on PCGS cannot be directly applied to other regions because the prevalence of AMR may be different between regions. Fourth, PCGS results were not compared with Gram staining results from the microbiology laboratory because of data insufficiency. Fifth, PCGS quality control was not evaluated in this study, although the utility of PCGS has been confirmed in several other studies from OCH [7, 8, 14, 25].

## Conclusions

Using PCGS with U/A led to a more precise fUTI diagnosis and encouraged clinicians to select narrower-spectrum antibiotics that showed high susceptibility. Urine PCGS could suppress the overuse of broad-spectrum antimicrobials, and it is advisable to include PCGS in all fUTI antimicrobial stewardship programs in adolescents and adults.

## Supplementary Information

**Additional file 1.** Original dataset.

## Data Availability

The dataset supporting the conclusions of this article is included within the article and its Additional file 1.

## Abbreviations

AMR: Antimicrobial resistance
CFU: Colony-forming units
CI: Confidence interval
CVA: Costovertebral angle
ER: Emergency room
ESBL: Extended-spectrum beta-lactamase
fUTI: Febrile urinary tract infection
GNR: Gram-negative rods
GPC: Gram-positive cocci
HPF: High power field
IDSA: Infectious Diseases Society of America
IQR: Interquartile range
OCH: Okinawa Chubu Hospital
PCGS: Point-of-care Gram stain
PSA: Prostate-specific antigen
U/A: Urinalysis
U/C: Urine culture
UTI: Urinary tract infection
WBC: White blood cell

## References

[1] Johnson JR, Russo TA (2018). Acute pyelonephritis in adults. New Engl J Med.

[2] van Nieuwkoop C, van der Starre WE, Stalenhoef JE, van Aartrijk AM, van der Reijden TJ, Vollaard AM (2017). Treatment duration of febrile urinary tract infection: a pragmatic randomized, double-blind, placebo-controlled non-inferiority trial in men and women. BMC Med.

[3] Wilson ML, Gaido L (2004). Laboratory diagnosis of urinary tract infections in adult patients. Clin Infect Dis.

[4] Grigoryan L, Trautner BW, Gupta K (2014). Diagnosis and management of urinary tract infections in the outpatient setting: a review. JAMA.

[5] Bennett JE, Blaser MJ (2019). Mandell, Douglas, and Bennett's principles and practice of infectious diseases.

[6] Kuijper EJ, van der Meer J, de Jong MD, Speelman P, Dankert J (2003). Usefulness of Gram stain for diagnosis of lower respiratory tract infection or urinary tract infection and as an aid in guiding treatment. Eur J Clin Microbiol Infect Dis.

[7] Taniguchi T, Tsuha S, Shiiki S, Narita M (2015). Gram-stain-based antimicrobial selection reduces cost and overuse compared with Japanese guidelines. BMC Infect Dis.

[8] Yodoshi T, Matsushima M, Taniguchi T, Kinjo S (2019). Utility of point-of-care Gram stain by physicians for urinary tract infection in children ≤36 months. Medicine.

[9] Schaeffer AJ, Nicolle LE (2016). Urinary tract infections in older men. N Engl J Med.

[10] Taniguchi T, Tsuha S, Shiiki S, Narita M (2018). High positivity of blood cultures obtained within two hours after shaking chills. Int J Infect Dis.

[11] Rubin RH, Shapiro ED, Andriole VT, Davis RJ, Stamm WE (1992). Evaluation of new anti-infective drugs for the treatment of urinary tract infection. Infectious Diseases Society of America and the Food and Drug Administration. Clin Infect Dis.

[12] Ulleryd P, Zackrisson B, Aus G, Bergdahl S, Hugosson J, Sandberg T (1999). Prostatic involvement in men with febrile urinary tract infection as measured by serum prostate-specific antigen and transrectal ultrasonography. BJU Int.

[13] Kiyoyama T, Tokuda Y, Shiiki S, Hachiman T, Shimasaki T, Endo K (2009). Isopropyl alcohol compared with isopropyl alcohol plus povidone-iodine as skin preparation for prevention of blood culture contamination. J Clin Microbiol.

[14] Taniguchi T, Tsuha S, Shiiki S, Narita M (2020). Point-of-care cerebrospinal fluid Gram stain for the management of acute meningitis in adults: a retrospective observational study. Annal Clin Microbiol Antimicrob.

[15] Sorensen JB, Klee M, Palshof T, Hansen HH (1993). Performance status assessment in cancer patients. An inter-observer variability study. Br J Cancer.

[16] Cantey JB, Gaviria-Agudelo C, McElvania TeKippe E, Doern CD (2015). Lack of clinical utility of urine Gram stain for suspected urinary tract infection in pediatric patients. J Clin Microbiol.

[17] Anchinmane VT, Preet K, Sankhe S (2018). Utility of urinary gram stain as a diagnostic method for urinary tract infection. Int J Res Med Sci.

[18] Wiwanitkit V, Udomsantisuk N, Boonchalermvichian C (2005). Diagnostic value and cost utility analysis for urine Gram stain and urine microscopic examination as screening tests for urinary tract infection. Urol Res.

[19] Franco-Paredes C, del Rio C, Jurado R (2002). The clinical utility of the urine Gram stain. Infect Dis Clin Pract.

[20] Czaja CA, Scholes D, Hooton TM, Stamm WE (2007). Population-based epidemiologic analysis of acute pyelonephritis. Clin Infect Dis.

[21] Grillo S, Cuervo G, Carratalà J, Grau I, Llaberia M, Aguado JM (2020). Characteristics and outcomes of *Staphylococcus aureus* bloodstream infection originating from the urinary tract: a multicenter cohort study. Open Forum Infect Dis.

[22] Gupta K, Hooton TM, Naber KG, Wullt B, Colgan R, Miller LG (2011). International clinical practice guidelines for the treatment of acute uncomplicated cystitis and pyelonephritis in women: a 2010 update by the Infectious Diseases Society of America and the European Society for Microbiology and Infectious Diseases. Clin Infect Dis.

[23] Doi A, Shimada T, Harada S, Iwata K, Kamiya T (2013). The efficacy of cefmetazole against pyelonephritis caused by extended-spectrum beta-lactamase-producing Enterobacteriaceae. Int J Infect Dis.

[24] Japan Nosocomial Infections Surveillance (JANIS) Annual Open Report. 2015. https://janis.mhlw.go.jp/english/report/index.html. Accessed 6 Dec 2021.

[25] Fukuyama H, Yamashiro S, Kinjo K, Tamaki H, Kishaba T (2014). Validation of sputum Gram stain for treatment of community-acquired pneumonia and healthcare-associated pneumonia: a prospective observational study. BMC Infect Dis.

